# Considerations for Maximizing the Exercise “Drug” to Combat Insulin Resistance: Role of Nutrition, Sleep, and Alcohol

**DOI:** 10.3390/nu13051708

**Published:** 2021-05-18

**Authors:** Mary-Margaret E. Remchak, Kelsey L. Piersol, Sabha Bhatti, Andrea M. Spaeth, Jennifer F. Buckman, Steven K. Malin

**Affiliations:** 1 Department of Kinesiology & Health, Rutgers University, New Brunswick, NJ 08901, USA; mary.remchak@rutgers.edu (M.-M.E.R.); klp183@scarletmail.rutgers.edu (K.L.P.); ams853@kines.rutgers.edu (A.M.S.); jbuckman@rutgers.edu (J.F.B.); 2 Division of Cardiovascular Medicine, Rutgers University, New Brunswick, NJ 08901, USA; sb1702@rwjms.rutgers.edu; 3 Center of Alcohol Studies, Rutgers University, Piscataway, NJ 08854, USA; 4 Division of Endocrinology, Metabolism & Nutrition, Rutgers University, New Brunswick, NJ 08901, USA; 5 New Jersey Institute for Food, Nutrition and Health, Rutgers University, New Brunswick, NJ 08901, USA; 6 Institute of Translational Medicine and Science, Rutgers University, New Brunswick, NJ 08901, USA

**Keywords:** cardiovascular disease, endothelial function, glycemic control, physical activity, circadian rhythm, diet

## Abstract

Insulin resistance is a key etiological factor in promoting not only type 2 diabetes mellitus but also cardiovascular disease (CVD). Exercise is a first-line therapy for combating chronic disease by improving insulin action through, in part, reducing hepatic glucose production and lipolysis as well as increasing skeletal muscle glucose uptake and vasodilation. Just like a pharmaceutical agent, exercise can be viewed as a “drug” such that identifying an optimal prescription requires a determination of mode, intensity, and timing as well as consideration of how much exercise is done relative to sitting for prolonged periods (e.g., desk job at work). Furthermore, proximal nutrition (nutrient timing, carbohydrate intake, etc.), sleep (or lack thereof), as well as alcohol consumption are likely important considerations for enhancing adaptations to exercise. Thus, identifying the maximal exercise “drug” for reducing insulin resistance will require a multi-health behavior approach to optimize type 2 diabetes and CVD care.

## 1. Introduction

Cardiovascular disease (CVD) has remained the leading cause of death above all other major diagnostic groups for the better part of a decade [1]. CVD generally refers to myocardial infarction, heart failure and stroke that affect the blood vessels. Deaths from CVD worldwide are projected to increase from 17.8 million in 2017 to more than 22.2 million by 2030 [1]. Einarson et al. [2] reported that CVD development is also considered a comorbidity to the progression of type 2 diabetes mellitus (T2DM), with nearly 34.2 million individuals in the U.S. diagnosed with T2DM, and another 88 million adults with prediabetes [3]. Concerningly, nearly 32.2% of all persons with T2DM have CVD [2]. A common link between T2DM and CVD is insulin resistance, or low insulin sensitivity [4]. Insulin resistance is defined as an inability of insulin to exert several effects on key organs to maintain normoglycemia. This can occur due to either insulin receptor defects (e.g., receptor protein content) and/or impaired insulin signal transduction [5]. If insulin secretion fails to compensate adequately for defects in insulin action, regardless of the exact cellular mechanism, then hyperglycemia develops and further promotes oxidative stress and inflammatory responses that can result in vascular damage [4]. Additionally, insulin resistance/hyperinsulinemia is associated with dyslipidemia (i.e., low HDL and high VLDL/LDL cholesterol/triglycerides (TG)) which are also potential triggers for endothelial dysfunction and atherosclerotic plaque formation [4,6,7].

The American Heart Association (AHA) [1] provides a good framework in promoting regression, delayed onset, and/or prevention of CVD. Referred to as “Life’s Simple 7”, the AHA advises that individuals maintain the following: (1) systolic/diastolic blood pressure ratio of <120/80 mmHg, (2) total cholesterol (TC) <200 mg/dL, (3) fasting glucose <100 mg/dL, (4) no smoking, (5) body mass index (BMI) <25 kg/m^2^, (6) diet score meeting 4–5 target components (to consume ≥4.5 cups/d fruits and vegetables, ≥2 servings/w of fish, ≥3 servings/d of whole grains, ≤36 oz/w of sugar-sweetened beverages, and ≤1500 mg/d of sodium), and (7) live a physically active lifestyle [8]. Although sufficient physical activity (≥3.5 h/w aerobic or functional exercise) is independently linked to reduced mortality [6], being physically active in combination with a healthy diet (e.g., Mediterranean Diet Score ≥5) that meets AHA recommendations along with moderate alcohol consumption (≤2 drinks/d for men and ≤1 drink/d for women), and not smoking can reduce risk of composite CVD and risk fatal CVD by 57 to 67% [9]. Furthermore, the inclusion of sufficient sleep (≥7 h) to these healthy lifestyle factors is associated with 65% lower risk of composite CVD and 83% lower risk of fatal CVD [9]. Thus, a key challenge for many scientists and healthcare providers is determining the optimal “dose” for each component that works in synergy to maximize CVD risk reduction.

From this perspective, exercise could be viewed as a “drug” and be considered in a similar manner as traditional pharmaceutical drugs (i.e., exogenous stimuli) intended to prevent and/or treat disease via physiological adaptations of bodily structure and function [10]. To provide the right prescription for health gains, healthcare providers need to consider the dose, type, and timing in which exercise is provided. Further, nutrient intake, sleep and/or alcohol consumption surrounding exercise may be critical for optimizing the beneficial health gains through improved insulin sensitivity and reduced CVD risk [11]. The ideal exercise prescription though remains unclear. This review will focus on the pathophysiology of insulin resistance as a chief underlying factor associated with CVD risk. Specifically, we will mention the influence of exercise “dosing” (i.e., intensity, frequency, duration, and modality), with particular attention placed on exercise timing (e.g., morning vs. night and breaks in sedentary behavior) as well as the proximal food intake (e.g., fasted/fed exercise, etc.). The possible ways that sleep and alcohol intake could independently influence the effectiveness of exercise to lower insulin resistance will also be discussed. Lastly, we will conclude with clinical perspective on considering such behaviors for maximizing the exercise “drug”.

## 2. Insulin Resistance as a Link to CVD

Insulin is a key endocrine hormone that regulates glucose homeostasis during the fasting and fed state [12]. Insulin secretion from pancreatic β-cells is primarily stimulated in response to elevated post-prandial blood glucose, although nutrients like amino acids can have secondary effects [12]. Insulin acts largely on skeletal muscle to promote glucose uptake [13] and the liver to suppress production [14]. Insulin also can inhibit lipolysis, thereby reducing circulating free fatty acid (FFA) to foster normoglycemia. However, for insulin to induce these metabolic effects, it is important to acknowledge that insulin promotes blood flow to allow adequate oxygen, hormones, and nutrients as well as clearance of waste [15]. Insulin resistance can raise CVD incident following the “common soil” hypothesis due to shared mechanisms (e.g., oxidative stress/inflammation) [16]. 

### 2.1. Tissue Regulation of Insulin

#### 2.1.1. Skeletal Muscle and Insulin

While the role of individual tissues (e.g., skeletal muscle, liver, adipose, etc.) contributing to systemic glucose control has been previously reviewed [5], it is important to acknowledge that skeletal muscle is responsible for nearly 80% of insulin-stimulated whole-body glucose uptake [12,17,18]. AMP-activated protein kinase (AMPK) is activated via skeletal muscle contractions in response to energy depletion, thereby contributing, at least partially, to enhancing GLUT-4 regulation [19]. Importantly, for nutrients to be taken up by skeletal muscle, insulin must promote glucose uptake by stimulating GLUT-4 via a phosphatidylinositol 3-kinase (PI3-K) to AS160 mediated mechanism [20]. This shift towards glucose uptake favors either primarily oxidation and/or storage since insulin stimulation suppresses lipolysis and reduces fasting fuel preferences of fat utilization [18]. This active shift in substrate utilization from fasting fat oxidation to insulin-stimulated glucose reliance is referred to as metabolic flexibility [21]. Insulin resistance is associated with metabolic inflexibility, highlighting that altered mitochondrial function may contribute to reduced insulin sensitivity via reduced oxidative capacity [22].

#### 2.1.2. Liver and Insulin

The liver is primarily involved in glucose production during the fasted state due to high glucagon and low insulin levels [23,24]. This source of glucose production originates from either glycogenolysis [24] and/or gluconeogenesis, with the latter being considered the primary defect in progression to T2DM [25]. In contrast, during the fed state insulin acts on the liver with concomitant reductions in circulating glucagon to take up glucose for synthesis of glycogen and suppress hepatic glucose production [24]. Moreover, the liver also regulates lipid homeostasis by increasing de novo lipogenesis, suppressing fatty acid oxidation, and promoting the esterification and secretion of TG [24,26]. Additionally, fatty acid stimulated VLDL play a primary role of hepatic derived TG transportation from the liver to adipose tissue for storage via lipoprotein-mediated hydrolysis when energy is needed [27]. Alternatively, when energy is in low demand VLDL can be re-esterified to glycerol and stored as fat [27]. Not surprisingly, hepatic insulin resistance presents as an inability to properly stimulate glucose uptake and suppress hepatic glucose production as well as represents an important mechanism in the development of nonalcoholic fatty liver disease (NAFLD) [28].

#### 2.1.3. Adipose Tissue and Insulin

Adipose tissue is not only the main organ for TG storage, but also an important endocrine organ that influences the glucose and lipid metabolism through its release of adipokines, proinflammatory factors, and FFA. In turn, adipose tissue is responsible for roughly 10–20% of whole-body glucose utilization through influences on insulin signaling [29,30]. Indeed, since insulin has a direct effect on adipose tissue to promote glucose uptake and TG synthesis and suppress the release of FFA and glycerol into circulation [29,30,31], it is not surprising that adipose insulin resistance is associated with the onset of skeletal and hepatic insulin resistance [32]. Furthermore, disrupted adipocyte function in the ability to store excess TG is a precursor for systemic insulin resistance [33]. Decreased TG storage capacity within adipose tissue also creates an increased flux of plasma FFA to surrounding tissues thereby promoting fat redistribution and ectopic fat deposition (e.g., visceral fat, hepatic steatosis, etc.). This ectopic fat is clinically concerning since it is associated with reduced insulin-mediated glucose disposal in skeletal muscle [33] and endothelial function [34]. Assessment of cultured adipocytes have shown that increased FFAs also increase oxidative stress that, in turn, augments proinflammatory cytokine responses that contribute to the initiation and progression of atherosclerosis [35]. Collectively, insulin resistant adipose tissue can subsequently lead to improper TG synthesis and storage, as well as create a compounding effect in the development of CVD and associated risk.

#### 2.1.4. Vasculature and Insulin

The vascular component of insulin action has received less attention than aforementioned tissues, though it is still highly important. Indeed, insulin infusion in healthy individuals increases vasodilation of the femoral artery and plays a key role (e.g., up to 40%) in skeletal muscle glucose disposal [36,37]. In addition, insulin enhances the compliance of conduit arteries, relaxes resistance arterioles increasing tissue blood flow, and dilates precapillary arterioles for expansion of muscle microvasculature blood volume [38]. These vasodilatory actions of insulin are mediated through PI3K-dependent signaling pathways that initiate the production of nitric oxide (NO) from the endothelium [39,40]. NO, in turn, mediates multiple metabolic and vasculature processes (i.e., insulin delivery, resistance, and terminal arterioles dilation) that supports maintenance of blood glucose and lipids [41]. However, the inability to properly respond to insulin is related to endothelial dysfunction as well as arterial stiffness [42], which likely contribute to the development of CVD, atherosclerosis, T2DM, and metabolic syndrome (MetS) [38,43,44].

## 3. Exercise Treatment of Insulin Resistance

Exercise recommendations by the American College of Sports Medicine (ACSM) and the American Diabetes Association (ADA) [45] include engagement of ≥150 min/w of moderate-to-vigorous intensity aerobic activity (MVPA) spread over at least 3 d/w, with no more than two consecutive days without activity to lower risk of cardiovascular complications and overall mortality in T2DM [3]. It is also advised that 2–3 d/w of resistance exercise on nonconsecutive days be performed to promote acts of daily living/function [45]. Furthermore, all adults are recommended to decrease time per day of sedentary behavior with prolonged sitting interrupted every 30–60 min for peak blood glucose benefits, as well as engagement in flexibility and balance training 2–3 times/w [45]. Prolonged adherence to recommended exercise guidelines is associated with greater cardiorespiratory fitness and reduced incidence of T2DM [46,47]. Unfortunately, low participation and poor adherence to generalized exercise engagement is observed at high rates in many adults [48]. Thus, there is a need for more individualized exercise programs to maximize effectiveness and engagement. Using the FITT principle (i.e., frequency, intensity, time, and type) [48], we encourage readers to review work on exercise intensity and time (i.e., duration) which has been discussed extensively elsewhere [49,50,51]. Of note, while high-intensity interval training (HIIT) improves insulin resistance (via HOMA-IR assessment) in MetS patients [52], several studies show that when energy expended is matched between low to high exercise intensity conditions, no additional effects are observed on insulin action or endothelial function [53,54,55,56,57]. This suggests that differences in energy balance, not intensity per se, may account for such greater improvements in insulin sensitivity. Thus, the scope of this review will focus instead on less discussed topics including resistance exercise, exercise frequency/breaks in sedentary behavior, and the timing of exercise with proximal nutrient intake to consider more contemporary avenues that may improve the exercise “drug”. It is worth noting that discussions herein too blend both acute and chronic exercise. Acute exercise bouts are understood to improve insulin action via varied mechanisms from that of chronic exercise (e.g., muscle AMPK activity [19] vs. protein synthesis/fiber adaptations [48]). Because many view chronic exercise as reflection of accumulated single bouts, our attention will be placed on these considerations for defining the maximally effective exercise “drug” for health benefit.

## 4. Aerobic and/or Resistance Exercise for CVD Risk

Although there is substantial evidence that aerobic exercise leads to beneficial adaptations in insulin resistant individuals, there are fewer studies directly comparing aerobic to resistance exercise alone or in combination. This is a major knowledge gap since CVD is the leading cause of death and understandings of long-term exercise benefit on reducing CVD mortality and remains in question for people with T2DM [58]. Well documented studies such as the DARE [59], STRRIDE [60], and HART-D [61] clinical trials have examined how these exercise modalities compare to assess the most beneficial modality for combating T2DM and CVD risk factors. Together, they have provided evidence that the combination of aerobic and resistance exercise induces greater reductions in HbA1c [59,61,62] and central adiposity/percent body fat [61,62], as well as improved peak oxygen consumption (VO_2peak_) [61] compared with either aerobic or resistance alone. However, an important consideration when interpreting this work is that the DARE (aerobic: 15–20 min at 60% HRmax progressed to 45 min at 75% HRmax, resistance: eight weight bearing exercises with progressive load increase, 2–3 sets with eight rep max, combination: aerobic + resistance full programs) [63] and STRRIDE (aerobic: low-volume/moderate intensity (40–55% VO_2peak_) and high intensity (65–80% VO_2peak_) exercise of 12 miles/w, 1200 kcal/w equivalent to 14 kcal/kg/w, and high-volume/high intensity exercise of 20 miles/w, 2000 kcal/w at 65–80% VO_2peak_ equivalent to 23 kcal/kg/w for a 90 kg person) [60] studies did not equate work load or energy expenditure between exercise conditions. This is problematic because the combined exercise groups did twice the workload of aerobic or resistance exercise only, thereby raising questions to whether concurrent exercise is better than either one alone. Indeed, dose-response improvements in insulin resistance have been reported following exercise, with increasing energy expenditure related to better outcomes [64,65,66]. The HART-D trial (aerobic: 50–80% VO_2max_ for 12 kcal/kg/w, resistance: 2–3 sets of 9 whole body exercises for 10–12 reps, combination: 10 kcal/kg/w aerobic + 2 sessions of one set per nine above mentioned exercises resistance) though is one of the few studies to date that attempted to match weekly training kcal expenditure across exercise groups [61]. When aerobic versus resistance exercise was compared to the combination of aerobic + resistance exercise at equivalent energy expenditure conducted 3–5 d/w over a 9 month period, all modalities reduced total fat, visceral adiposity, and ectopic liver fat, intrahepatic as well as skeletal lipids [61,62]). Recently, substitution of a portion of total HIIT duration of aerobic training (i.e., stationary bike) by resistance training (i.e., squat, lung, and deadlift) in insulin resistant MetS patients also suggested improved blood glucose metabolism, VO_2max_, and body composition (i.e., body weight, fat mass, and waist circumference) collectively when compared to aerobic based HIIT at matched exercise duration and energy expenditure [52]. Interestingly, though HIIT exercise only reduced TG and blood pressure when compared with this HIIT and resistance exercise program [52,67]. Although the STRIDDE [68] trial suggested that aerobic plus resistance exercise lowered metabolic syndrome severity (i.e., z-score) more than aerobic alone, the collective work suggests that weight lifting may have unique CVD risk factor effects compared with that of aerobic exercise [66]. Additional work is warranted testing the impact of aerobic plus resistance on CVD risk since none of the aforementioned studies were specifically designed to examine endothelial function and/or arterial stiffness and these are independent markers of CVD risk.

## 5. Breaking up Inactivity with Exercise

Prolonged sitting is adversely associated with premature CVD, abnormal blood glucose and lipid metabolism, and all-cause mortality independent of MVPA [69,70]. In addition, sedentary behavior may further influence increased energy intake as well as screen time [69] during the accumulating day of sitting in the average adult, thereby promoting excess weight gain. This is concerning since over 75% of working hours are sedentary [71]. In fact, 1 extra hour of sedentary time over an 8 d period is associated with a 22% increased odds of T2DM and a 39% increased odds of MetS in those between the ages of 45–70 [72]. Further, sedentary behavior is related to hyperglycemia independent of aerobic fitness [73], although high fitness can attenuate cardiovascular risk factor clustering [74].

Interrupting extended bouts of sitting or sedentary behavior with frequent breaks of exercise is associated with improved cardiometabolic risk factors and decreased all-cause mortality risk [70]. Breaking up inactivity with 2 min bouts of light-intensity walking every 20 min for 5 h during the postprandial phase reduced both glycemic and insulinemic responses, with similar positive outcomes observed at moderate-intensity walking [69,70]. In adults with T2DM, the interruption of prolonged sitting with light intensity walking or simple resistance activities (e.g., half-squats, knee/calf raises, and gluteal contractions) for 3 min every 30 min over 8 h decreases postprandial glucose, insulin, C-peptide, and triglyceride levels [75]. Intermittent stair climbing too as a form of activity to break up sedentary behavior for about 60–180 s of brisk walking has also been effective at reducing blood glucose in some [76,77], but not all studies for 1 d or up to 6 w in T2DM [78]. Interestingly, replacing sitting time with standing (2.5 h/d) and light-intensity walking (total 2.2 h/d) preferably every 30 min improved 24 h blood glucose levels and insulin sensitivity in individuals with T2DM to a greater extent than structured exercise [79]. In males with T2DM, 3 × 15 min bouts of activities (175 kcal/each) for daily living post-meal were effective at lowering blood glucose and insulin, but only continuous moderate intensity exercise (50% Wmax; 350 kcal) significantly reduced the time exposed to hyperglycemia (i.e., >10 mM) [80]. This is consistent with recent work in “free-living” environments vs. that of the lab. Recent work suggests implementation of breaks in sitting with physical activity may be challenging in “free-living” environments, as only continuous walking was shown to reduce daily hyperglycemia exposure [81]. Regardless, 5 min breaks over 12 h were shown to more effectively lower blood glucose and insulin production rate than 1 continuous bout for 60 min at 65% VO_2max_ in people with impaired glucose tolerance [82]. In line with this work, others have shown that breaks in sedentary behaviors affects endothelial function as well. Indeed, Taylor et al. [83] showed that breaking up sedentary behavior with resistance exercise every 30 min, compared to 60 min, in people with T2DM increased femoral artery flow-mediated dilation (FMD) over 7 h while compared with uninterrupted sitting. Interestingly, in non-obese young men, performing light physical activity on the hour for 3 h prevented the decline in femoral FMD [84]. These acute effects seem to translate over 16 wks in older overweight adults as well, whereby mobile health intervention to break up sedentary behavior resulted in a 1 h reduction in sedentary time (about 10 to 9 h) with light breaks in physical activity related to femoral FMD as well as cerebrovascular blood flow [85]. Collectively, these findings support the idea that breaking up sedentary behavior by accumulating small “doses” of exercise throughout the day can have clinically meaningful impacts on CVD risk vs. routine continuous exercise. Additional work examining insulin resistance is warranted to understand if long-term use of breaks in sedentary behavior can benefit chronic disease.

## 6. Clinical Nutrition and Insulin Resistance

Chronic energy surplus is strongly linked to the development of obesity and associated CVD risk factors [86]. Further, dietary habits that include high-fat and refined-carbohydrate ingestion, and central adiposity are directly related to the development of insulin resistance [52]. Current nutritional recommendations for individuals at risk for CVD range depending on the specific underlying pathology. For instance, people with insulin resistance are often advised to lose about 5–10% body weight with emphasis on restriction of calories (e.g., saturated fats to <7% of total energy intake, <200 mg/d cholesterol intake, restriction of trans fat intake) and consumption of high-fiber intake of at least 14 g/1000 kcal [86]. In fact, several studies support that a caloric restriction program, with or without exercise, is effective at improving blood glucose, insulin sensitivity, endothelial function and/or arterial stiffness [87,88,89,90,91], although the combination of diet plus exercise may be best given gains in fitness in lieu of energy deficit being matched [91,92,93]. Other possible dietary approaches to reduce CVD risk include the DASH [94,95,96], Mediterranean [97,98,99], and carbohydrate-restricted vs. low-fat diets [100]. Although a common theme to many of these dietary approaches for reducing CVD risk stem from either a weight loss standpoint and/or changes in macronutrient content that attenuate compensatory hyperinsulinemia seen with insulin resistance [86], it is important to highlight that few data exist systematically testing these diets in combination with exercise under matched energy balance conditions. We encourage readers to refer elsewhere [101] that discusses various diets and their interaction with exercise for glycemic/CVD risk benefit.

## 7. Exercise Timing with Proximal Nutrition

An important consideration with exercise timing centers around meals. If someone exercises and does not consume calories to replace energy expended, a person will be in an energy deficit and create negative energy balance. To offset this, a fair question is: should we exercise fasted or in the post-prandial state for clinical benefit? In general, many people when exercising might consider consuming food/supplements/beverages (e.g., carbohydrate based) to support their movement. While there is little doubt consumption of carbohydrate aids performance in healthy, active people, the benefits of consuming calories in sedentary individuals are less clear. This said, we know that mouth rinse and spit approaches of carbohydrate during exercise enables athletes to increase athletic performance [102]. Whether rinse and spit carbohydrate solutions should be used as a method for improved exercise adherence or perception remains to be fully worked out. Regardless, for this review, the question of whether nutrient ingestion during exercise helps people with chronic disease risk and if it is important for metabolic adaptations since nutrient intake suppresses fat oxidation/adaptations related to insulin sensitivity warrants attention [103]. Aerobic exercise often lowers muscle glycogen, thereby fostering enhanced ability for insulin to promote glucose uptake for synthesis of glycogen [104]. Thus, exercising fasted may yield greater benefit. It was shown that pre-meal “exercise snacking” (6 × 1 min intense incline walking at 90% HRmax on a treadmill) 30 min before a meal reduced blood glucose at breakfast and dinner compared to no exercise or a single 30 min bout of moderate intensity walking 30 min prior to dinner (60% HRmax) [105]. In contrast, in adults with prediabetes, 3 sets of 15 min bouts at 3 METs after a meal lowered post-prandial blood glucose better than 45 min of continuous morning or afternoon exercise [106]. Furthermore, resistance exercise (up to 40% of their bodyweight; e.g., leg press, seated chest flyes, shoulder raises, etc.) conducted after dinner lowered glucose and TG the following morning compared with pre-dinner resistance exercise that lowered only glucose in people with T2DM [107]. A recent systematic review concluded that postprandial aerobic exercise compared to non-exercise control decreased short-term glucose area under the curve by upwards of 26% and 24 h prevalence of hyperglycemia by upwards of 65%, whereas resistance exercise decreased glucose area under the curve by 30% and 24 h prevalence of hyperglycemia by 35% [108]. Indeed, a brief periodic exercise (3 sets of 1-min light intensity jogging + 30 s of rest; total duration of 4 min for each exercise bout, every 30 min throughout the day, 20 times in total) may be more effective at blunting post prandial glucose (PPG) compared with pre-exercise, particularly at breakfast in relatively active, healthy males [109]. These collective findings suggest “exercise snacking” is effective, but that post-prandial timing of this activity may yield more benefit for attenuating both glycemia and TG concentrations. Whether long-term training adaptations are indeed better, remain to be thoroughly investigated. In fact, over the last several years, work has suggested that training in the fasted state could yield greater mitochondrial adaptations [110]. Moreover, others have suggested that training in the fasted state for just 6 w yielded greater insulin sensitivity benefit during an oral glucose tolerance test (OGTT) compared to when obese individuals trained in the fasted state [111]. However, timing in which exercise was performed was not systematically controlled in these former studies. This could be important as new work suggests training in the morning fasted, compared to exercise after breakfast, yielded greater exercise fat oxidation as well as insulin sensitivity gains [112] in overweight/obese adults. In contrast, in people with T2DM there was no added benefit for HbA1c when training aerobically for 12 w in either the fasted or fed state [113]. Consequently, additional work is needed to definitively show if proximal nutrient intake has long-term clinical benefit despite the collective evidence suggesting post-prandial exercise at least acutely more favorable for glycemic end-points related to chronic disease.

Recent work has also begun to target the best time of day to exercise. This has evolved in part since circadian biology reveals people tend to fluctuate on a continuum throughout the day, and people appear to become glucose intolerant via lower insulin sensitivity and/or endothelial function in the evening compared with the morning [114,115]. As a result, it would be reasonable to hypothesize that exercise in the afternoon may be best at combating these declines in insulin sensitivity and/or endothelial function to optimize training adaptations. Indeed, a recent pilot study of high intensity interval exercise was shown to more effectively lower interstitial fluid glucose measured by continuous glucose monitoring when performed in the afternoon versus the morning [116]. Further, these data are supported in a recent retrospective study highlighting that skeletal muscle and adipose insulin sensitivity were improved to a greater extent when exercise was performed in the afternoon compared with morning [117]. However, not all studies agree that afternoon is the best time to exercise. Teo et al. [118] demonstrated that 12 w of aerobic plus resistance exercise improved glycemic control whether performed in the morning or afternoon in adults with T2DM. Additionally, varied responses by sex to exercise timing when adjustments for activity level were considered in adults with T2DM suggests men who performed midday MVPA presented with lower cardiorespiratory fitness, whereas those who performed more morning activity presented with the highest cardiorespiratory fitness [3]. In contrast, adult women with T2DM presented with higher cardiorespiratory fitness when engaging in evening MVPA versus morning or midday activity [3]. Somewhat paradoxical though was the observation of higher CVD risk as measured by the Framingham risk score in men who exercised in the morning despite this fitness relationship and no associations were noted in women. Various factors may be at play in this retrospective study [3], including nutrition/sleep/alcohol and further investigation from prospective studies are needed. Indeed, others show that progressive moderate intensity aerobic training (i.e., cycle ergometer) performed in treated hypertensive middle-aged men suggests evening exercise significantly decreases clinic and ambulatory blood pressure (24 h and asleep diastolic blood pressure (DBP)) via reduced systemic vascular resistance and vasomotor sympathetic modulation [119]. Taken together, more work is warranted to understand the best time of day to exercise given circadian influence may impact metabolic as well as vascular adaptation for CVD prevention/treatment.

## 8. Sleep and Relations to CVD Risk

Emerging evidence has identified sleep as an important behavioral target that contributes to chronic disease risk [120]. Insufficient sleep and poor quality sleep are common, with 33% of adults reporting habitual sleep durations <7 h/night and 32% complaining of frequent sleep disturbances [121,122]. Causes of poor sleep include work and family obligations, circadian misalignment (e.g., shift work), having an evening chronotype, and suffering from a clinical sleep disorder (with sleep apnea and insomnia being the most common) [121,123]. Population studies have demonstrated an association between poor sleep and increased risk for obesity, T2DM and CVD [120]. Laboratory studies have begun to elucidate potential mechanisms underlying the relationship between poor sleep and adverse cardiometabolic effects including decreased insulin sensitivity, endothelial dysfunction, increased blood pressure, weight gain, and attenuated fat-loss when dieting [120].

In addition to the interactions of poor sleep and CVD risk [124], it is also important to consider how sleep interacts with diet and exercise. Experimental sleep restriction (e.g., five consecutive nights of 4 h time-in-bed) leads to increases in: hunger, impulsivity in response to food cues, late-night eating, portion sizes, fat intake, and daily caloric intake [125]. Although less work has been done to examine how sleep restriction affects physical activity levels, there is evidence that individuals are more sedentary after a night of insufficient sleep [126]. Overall, sleep shares a positive bidirectional relationship with diet and exercise such that individuals who report sufficient, good quality sleep also report healthier dietary patterns (e.g., reduced late-night snacking, healthy portion sizes, lowered fat intake, and recommended daily caloric intake) and increased physical activity levels [127,128]. To date, unfortunately it is not clear if better sleep drives better eating or if healthy dietary patterns foster good sleep and this is an area needing more work. Regardless, this is important as adequate sleep is likely to create an environment whereby people can maximize the exercise effects on insulin action without experiencing accentuated stress responses, although the physiological mechanisms remain to be elucidated. For instance, acute sleep deprivation prior to a bout of exercise impacts performance such that individuals exhibit declines in Wmax and HRmax during a maximal incremental test [129], increased ratings of perceived exertion [130,131], decreased exercise minute ventilation and time to exhaustion [132] and impairment in maximal muscle strength during compound movements, potentially via reductions in motivation [133].

When measuring CVD risk factors, evidence suggests that exercise may also partially attenuate the adverse effects of sleep restriction. For example, a study examined blood glucose and insulin responses in men during two conditions across a 4 d experiment: sedentary daily activity and sleep deprivation (60 h) and daily physical activity (e.g., cycling for 20 min morning/mid-day followed by treadmill running in late afternoon at 70–80% VO_2max_ on day 1 and 3; 6 h hike day 2 at about 24% VO_2max_ on day 2) and sleep deprivation (60 h). In response to an OGTT, no differences in total plasma glucose response were observed; however, the insulin response was lower in the active versus sedentary condition [134]. A similar, more recent study, also in males, investigated the effects of HIIT on insulin resistance induced by sleep deprivation of 24 h. Participants experienced four conditions: regular sleep, sleep deprivation, HIIT training followed by regular sleep, and HIIT training followed by sleep deprivation. HIIT took place across six training sessions over 2 w and each session consisted of 8–12 × 60 s intervals at 100% of peak power output. In response to an OGTT, sleep deprivation increased glycaemia and insulin as well as increased FFA concentrations. However, HIIT prior to sleep deprivation attenuated the increase of these substrates, suggesting preservation of insulin action by exercise [135]. Furthermore, HIIE for 3 d has been reported to reverse the short-term deleterious effects of sleep deprivation (4 h time-in-bed) on muscle protein synthesis in young men [136]. This later finding is important as sleep restriction has been associated with loss of muscle mass, which is important for both basal metabolic rate as well as insulin sensitivity and overall daily function. From a CVD standpoint, a recent paper investigated differences between middle-aged adults who were chronic sufficient (≥7 h/night) or short sleepers (<7 h/night) on vascular function. At baseline, the short sleepers exhibited impairments in endothelial vasodilation as measured by forearm blood flow. However, aerobic exercise for 3 months improved measures of endothelial function (ACh-mediated vasodilation, ET-1-mediated vasoconstriction, and the vasodilator response to ACh with ETa receptor blockade) in the short sleepers [137]. These later findings suggest that exercise increases endothelial function, in part, by improving vasomotor tone (i.e., reduced vasoconstriction). Taken together, it seems that while exercise can “rescue” the deleterious effects of acute sleep deprivation, few data are available demonstrating how sleep impacts metabolic and vascular adaptations to exercise over time and further work is needed [138].

## 9. Alcohol Interactions with CVD Risk

When considering the effects of exercise on insulin resistance and CVD risk, it is critical to also consider the impact of habitual alcohol use on exercise adaptations. Alcohol is among the most widely used pharmacological agents with approximately 70% of US adults [139] using alcohol this past year [140]. This is of potential concern since alcohol use that exceeds the definition of moderate drinking (i.e., >1 drink for women or >2 drinks for men, per day [141]) often leads to increased blood pressure, heart rate, and myocardial wall thickness which occurs, at least partially, through the renin–angiotensin–aldosterone system [142]. Excessive drinking has also been shown to promote oxidative stress [142] and reduce NO bioavailability [143,144] that may point to reactionary cascades ultimately impairing whole-body insulin action. Furthermore, excessive alcohol intake can increase hepatic fat content that, in time, leads to dyslipidemia (e.g., elevated TG in blood) as well as cirrhosis [145]. However, current epidemiological literature supports a J-shaped relationship between alcohol and both T2DM [146] and CVD risk [147], in that moderate alcohol intake has been shown to reduce NAFLD prevalence in the general population [148]. Additionally, moderate alcohol ingestion appears to increase HDL levels and insulin sensitivity as well as decrease oxidized LDL levels [142], which, coupled with decreases in platelet reactivity and aggregability, decrease CVD risk [142,149]. Therefore, light to moderate drinking, understood to be more common in the U.S. [141], may not exert the same negative effects as excessive drinking on health and well-being.

There is growing evidence that consuming alcohol during recovery from resistance exercise may disrupt skeletal muscle hypertrophy. This impediment on the anabolic effects of exercise may occur by bunting protein synthesis [150] and/or decreasing testosterone responses [151] that typically follows muscle-damaging exercise. In fact, previous work conducted in healthy young-adult males assessing the effects of alcohol consumption (mimicking binge drinking by athletes) post-exercise suggests reduced rates of myofibrillar protein synthesis by 37%, and when co-ingested with protein by 24% following high-intensity combined (AT+RT) exercise [152]. These outcomes suggest proper nutrition post-exercise may not fully attenuate the alcohol induced suppression of anabolic response in skeletal muscle [152]. It is possible that too much alcohol consumed post-exercise could negatively impact blood glucose via acute insulin resistance, and a direct relationship between reduced muscle mass and insulin-stimulated skeletal muscle GLUT-4 activation [153]. Taken together, this suggests that alcohol consumption may broadly antagonize exercise adaptations by undermining muscle structure and function. However, research to date on healthy adults has not clearly established the dose of alcohol at which these changes occur, nor has it confirmed effects in people at risk for or with T2DM/CVD.

Lower skeletal muscle mass has been linked to prospective CVD risk, especially in men [154], even after controlling for socioeconomic status, diet, tobacco use, hypertension, hypercholesterolemia, and diabetes. Numerous diseases involve muscle dysfunction and wasting, including alcohol use disorders [155]. In addition, exercise is increasingly prescribed to promote healthy aging [156] and as disease intervention [157]. For example, resistance training in older adults with T2DM shows specific metabolic and general health benefits such as reductions in plasma HbA1_c_, systolic blood pressure, and diabetes prescription doses along with increases in muscle glycogen stores and lean body mass [158]. Whether the inhibitory actions of alcohol use would attenuate these exercise effects is unknown, as is the dose, frequency, and timing of alcohol consumption at which this would be observed.

An additional consideration when examining the relationship between alcohol and exercise is the impact of behavioral choices. For example, do exercise and non-problematic alcohol use compete for time and salience? If an individual has a free hour from daily responsibilities, will they choose to have a beer or go for a run? Interestingly, a meta-analysis from 2012 [159] suggests that, on average, drinking and exercise behaviors may be complementary rather than competitive; alcohol consumption and physical activity was positively related among adults of all ages. This is in keeping with evidence that regular, moderate drinkers, and even binge drinkers (≥5 standard drinks in 2 h in men, ≥4 standard drinks in 2 h in women), appear more physically active than nondrinkers [159]. Nonetheless, factors such as chronotype are worth studying in the context of exercise timing and program adherence. Numerous sociocultural influences also limit non-problematic alcohol use to evening hours, after the day’s responsibilities are done [160]. For individuals inclined to exercise in the evening, a daily conflict between drinking and exercise may ensue, which thus may reduce motivation for physical activity and impact exercise adherence in a subset of non-problematic drinkers. Studies linking exercise and chronotypes to insulin sensitivity [161] further point to a growing interest in the multiplicative effects of lifestyle factors, such as exercise, alcohol, and sleep, on health. In fact, beyond alcohol–exercise interactions and sleep–exercise interactions, sleep–alcohol interactions are also actively being studied [162].

Further noteworthy is evidence that young adults who regularly binge drink and engage in routine exercise (3 d/w) have lower brachial artery endothelial dependent vasodilation [163,164]. This further suggests that the alcohol–exercise interaction is complex and a systematic approach to deconstruct the physiological mechanisms, temporal relationships, and dose dependencies in both healthy and clinical populations is needed. Dose–response studies must consider the interactive effects of individual bouts of exercise and alcohol consumption on health outcomes, as well as the cumulative effects from repeated exposures. Furthermore, the frequency, intensity, and type of exercise along with the quantity, frequency, and rate of alcohol consumed may induce differential effects on metabolic health [165], especially when considering timing between drinking and exercising. Of particular importance may be the implications of exercising during hangover when the cardiovascular system is already under stress and there is an increased risk for sudden cardiac death [166]. Comprehensive answers to these basic questions are not fully understood. Moderate drinking may be a ‘typical’ alcohol use behavior in of itself, but as seen with nutrition as well as sleep, varying levels of alcohol ingestion may counteract cardioprotective functions by direct vascular influences [142,167]. Taken together, the physiological and behavioral impacts of alcohol consumption, and, almost paradoxically, non-consumption, need to be considered to improve exercise “drug” prescriptions in adults of all ages, health statuses, and lifestyles.

## 10. Clinical Perspectives and Concluding Thoughts

**Figure 1:**
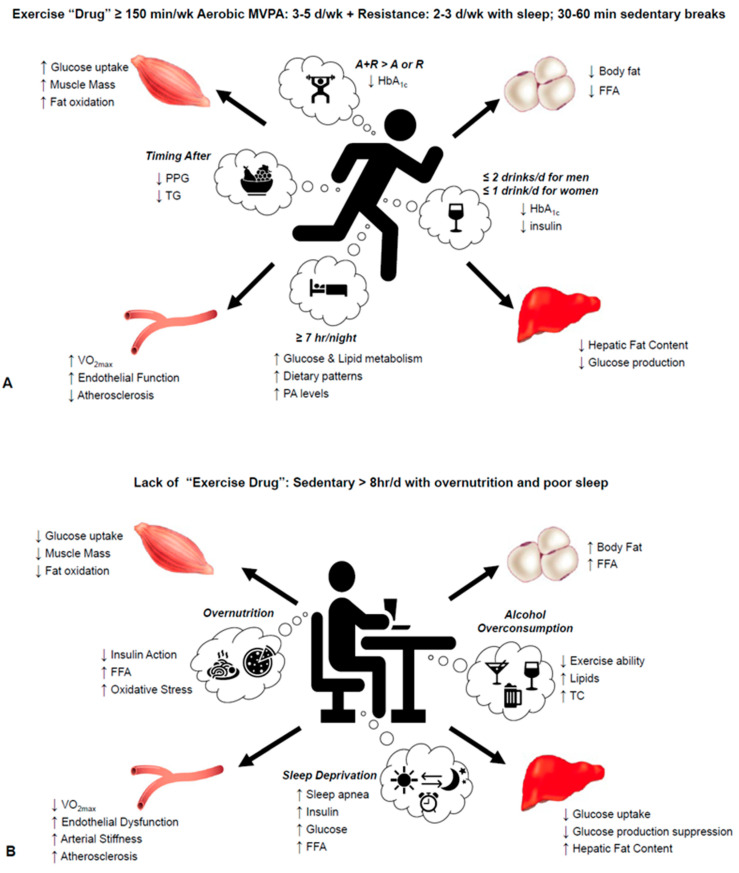
Considerations when designing the maximal exercise “drug” for insulin resistance and CVD risk reduction. (**A**) Adherence to the recommended exercise and health guidelines including sufficient amounts of sleep, moderate alcohol consumption, and diet focused on heart healthy foods associate with health. (**B**) Conversely, engagement in sedentary behaviors along with poor nutrition and sleep and excessive alcohol intake promotes CVD related risk. Aerobic exercise (A) performed 3–5 d/w; Resistance exercise (R) performed 2–3 d/w; HbA1c, hemoglobin A1c; PPG, post-prandial glucose; TG, triglyceride; VO_2max_, maximal aerobic capacity; PA, physical activity; MVPA, moderate vigorous physical activity; FFA, free-fatty acids; TC, total cholesterol.

For healthcare providers, educating patients about the maximal exercise “drug”, including considerations of nutrition, sleep and alcohol is challenging (Figure 1). Most of the literature suggests that accumulating 150 min/w of moderate to vigorous physical activity over 3–5 days (aerobic plus resistance exercise) is beneficial for cardiometabolic risk factor reduction. While many people will perform set periods of exercise (e.g., 30–60 min/session) throughout the week, it is fair to point out that accumulating the time may be equally, if not more effective, for some outcomes. Thus, breaking up sedentary behavior with physical activity independent of exercise is an important consideration in fostering maximal glycemic control, particularly after meals. Additionally, performing exercise fasted over time and, in the afternoon, may provide better results compared to when the same type of exercise conducted in the fed state and/or morning, although randomized clinical trials designed to impact insulin sensitivity are needed to confirm these considerations. Despite exercise being shown to have favorable effects on outcomes such as insulin sensitivity and/or endothelial function during inadequate sleep or alcohol consumption, receiving adequate sleep (about 7 h/night) as well as limiting alcohol consumption would likely support general health and dietary patterns focused on energy balance (including but not limited to; whole-grains and unprocessed foods with adequate fiber) to support overall CVD risk reduction. Regardless, for many sedentary patients lifestyle changes can be overwhelming and viewed as unrealistic given the time and money that may be required. In addition, many healthcare providers are not trained in behavior change education. Identifying though tangible goals with foundation in physical activity/exercise (e.g., breaking up sedentary behavior every 30 to 60 min with 1–2 min movements or walking for 15 min before/after a meal) is likely to provide a platform for systematically modifying other behaviors (e.g., diet, sleep, and alcohol) to support adherence to healthy behaviors. Another consideration though complicating these lifestyle behavior recommendations is that anti-diabetes, hypertension and lipid medications are often co-prescribed for CVD risk control. For example, the ADA recommends metformin as the first-line drug therapy [168]. If not successful in improving HbA1_c_, other agents such as, insulin secretagogues (e.g., sulfonylureas or GLP-1 agonists), DPPIV inhibitors (e.g., sitagliptin) or sodium-glucose cotransport inhibitors (e.g., canagliflozin) are indicated, with insulin injection therapy as an additional choice if glycemic targets are not met [168]. People at risk for or with T2DM are often prescribed other non-diabetes medications to manage comorbid conditions and these drugs can often impact lifestyle behaviors such as appetite, sleep quality, and/or adaptations to exercise [169,170,171,172,173]. Thus, while considerations for overall well-being must be made within context to design exercise maximally for treatment for T2DM and CVD prevention/management, it is important to remind people that it is never too late to take their first step.

## Data Availability

Not applicable.
